# NudC Deacetylation Regulates Mitotic Progression

**DOI:** 10.1371/journal.pone.0073841

**Published:** 2013-09-19

**Authors:** Carol Chuang, Jing Pan, David H. Hawke, Sue-Hwa Lin, Li-yuan Yu-Lee

**Affiliations:** 1 Department of Molecular and Cellular Biology, Baylor College of Medicine, Houston, Texas, United States of America; 2 Department of Medicine, Section of Immunology Allergy and Rheumatology, Baylor College of Medicine, Houston, Texas, United States of America; 3 Integrative Molecular and Biomedical Sciences, Baylor College of Medicine, Houston, Texas, United States of America; 4 Department of Translational Molecular Pathology, The University of Texas M.D. Anderson Cancer Center, Houston, Texas, United States of America; George Washington University, United States of America

## Abstract

Mitosis is largely driven by posttranslational modifications of proteins. Recent studies suggest that protein acetylation is prevalent in mitosis, but how protein acetylation/deacetylation regulates mitotic progression remains unclear. Nuclear distribution protein C (NudC), a conserved protein that regulates cell division, was previously shown to be acetylated. We found that NudC acetylation was decreased during mitosis. Using mass spectrometry analysis, we identified K39 to be an acetylation site on NudC. Reconstitution of NudC-deficient cells with wild-type or K39R acetylation-defective NudC rescued mitotic phenotypes, including chromosome misalignment, chromosome missegregation, and reduced spindle width, observed after NudC protein knockdown. In contrast, the K39Q acetylation-mimetic NudC was unable to rescue these mitotic phenotypes, suggesting that NudC deacetylation is important for mitotic progression. To examine proteins that may play a role in NudC deacetylation during mitosis, we found that NudC co-localizes on the mitotic spindle with the histone deacetylase HDAC3, an HDAC shown to regulate mitotic spindle stability. Further, NudC co-immunoprecipitates with HDAC3 and loss of function of HDAC3 either by protein knockdown or inhibition with a small molecule inhibitor increased NudC acetylation. These observations suggest that HDAC3 may be involved in NudC deacetylation during mitosis. Cells with NudC or HDAC3 knockdown exhibited overlapping mitotic abnormalities, including chromosomes arranged in a “dome-like” configuration surrounding a collapsed mitotic spindle. Our studies suggest that NudC acetylation/deacetylation regulates mitotic progression and NudC deacetylation, likely through HDAC3, is critical for spindle function and chromosome congression.

## Introduction

During mitosis, transcription is silent and RNA translation is globally inhibited. Mitosis is thus largely driven by posttranslational modifications of proteins, including phosphorylation [1], [2], methylation [3], [4], ubiquitination [5]–[7], and sumoylation [8]–[11]. As an example, histones are subject to a variety of modifications not only for transcriptional regulation, such as phosphorylation, methylation, ubiquitination and acetylation, not only for transcriptional regulation [12], [13], but also for executing mitotic events [13]–[16]. Recent proteomics studies suggest that protein acetylation is as prevalent as protein phosphorylation [17]–[20], implicating acetylation as an important mechanism in regulating cellular processes. In addition to histones, many non-histone proteins are also acetylated during mitosis [21]. Whether protein acetylation is involved in regulating the mitotic cell cycle has not been extensively studied.

We recently identified 51 non-histone proteins that are acetylated in mitosis [21]. These include proteins involved in cell cycle regulation, RNA translation and processing, chaperone function, DNA damage repair and metabolism. One of the acetylated proteins is nuclear distribution protein C (NudC), a highly conserved dynein/dynactin associated protein, which has been shown to play a role in mitosis and cytokinesis [22]–[24]. During mitosis, NudC plays a role in kinetochore-microtubule attachment, chromosome congression and spindle functions [23]. Whether NudC acetylation/deacetylation regulates NudC function in mitosis is not known.

Protein acetylation on lysine residues is mediated by histone acetyltransferases and is dynamically opposed by the actions of histone deacetylases (HDACs). Treatment of mitotic cells with histone deacetylase inhibitors was found to further increase the acetylation of a subset of mitotic proteins including NudC, suggesting that protein deacetylation in mitosis may be regulated by HDAC activity [21]. During mitosis, HDAC3, a member of the Class I HDACs, appears to be active [4], [25], [26]. HDAC3, together with the components of the N-CoR corepressor complex, is found to be localized on the mitotic spindle [25], [27]. Knockdown of HDAC3 or N-CoR exerted effects on mitotic spindle stability and the attachment of chromosomes to the mitotic spindle in a transcription-independent manner [25]. Treatment of mitotic cells with the HDAC inhibitor apicidin, which shows specificity for HDAC3 [28], also resulted in defects in spindle formation and chromosome organization on the mitotic spindle [21]. These observations suggest that the deacetylase activity of HDAC3 is required for proper mitotic progression. Whether HDAC3 activity is involved in the level of NudC deacetylation in mitosis is not clear.

In this study, we examined the role of NudC acetylation and deacetylation in mitosis. Our studies suggest that NudC acetylation was decreased during mitosis. HDAC3 is one of the enzymes that deacetylates NudC in mitosis and NudC deacetylation is critical for mitotic progression.

## Materials and Methods

### Antibodies and Histone Deacetylase Inhibitors

The following antibodies were used for immunoprecipitation (IP), blotting (dilutions shown), and immunofluorescence (IF; dilutions shown): acetyl-lysine (Millipore; rabbit, 0.5 µl/mg lysate for IP, 1∶2000), acetyl-lysine (Cell Signaling Technology; mouse, 1∶1000), H3 (Abcam; CHIP grade, rabbit, 1∶1000), HDAC3 (Millipore; 3G6, mouse, 1 µg/mg lysate for IP for this and all other antibodies), HDAC3 (GeneTex; C3, rabbit, 1∶2000), HDAC3 (GeneTex; N2C3, rabbit, 1∶2000 for IF), GFP (Sigma-Aldrich; mouse, 1 µg/mg lysate for IP), GFP (Invitrogen; rabbit, 1∶2000), NudC (Bethyl Laboratories Inc.; custom antibody, rabbit R2 against NudC C terminus 14 aa peptide, 1∶3000) [23], NudC (Bethyl Laboratories; custom antibody, goat G1 against NudC C terminus 14 aa peptide, 1∶2000 for IF) (see below), α-tubulin (GeneTex, rabbit, 1∶2000), α-tubulin (Sigma-Aldrich; rabbit, 1∶2000), β-tubulin(tub2.1) (Sigma-Aldrich, mouse, 1∶2000), and CREST serum (gift of Dr. Bill Brinkley, Baylor College of Medicine; human, 1∶5,000). Histone deacetylase inhibitors apicidin and sodium butyrate (NaB) were purchased from Sigma-Aldrich.

### Generation of Goat Anti-NudC Peptide Polyclonal Antibody

The goat G1 anti-NudC peptide polyclonal antibody was generated (contracted with Bethyl Laboratories) using the last 15 amino acids at the C-terminus of human NudC: DQHPEMDFSKAKFN. The same peptide was previously used to generate a rabbit anti-NudC peptide antibody [23]. The goat antibody was affinity-purified over an immunosorbent column to enrich for NudC-specific antibodies.

### Cell Culture and Synchronization

HeLa cells (ATCC CCL2) are cultured in DMEM supplemented with 10% fetal bovine serum (FBS) (Invitrogen). To enrich for mitotic (M) cells, HeLa cells were synchronized by double thymidine (Sigma-Aldrich) block (DTB) and release with nocodazole (Noc; Sigma-Aldrich) enrichment with or without 50 nM, 100 nM, or 500 nM apicidin treatments [21]. M cells were harvested by lightly pipeting with disposable transfer pipets (VWR). Asynchronous (Asy) cells were seeded concurrently and harvested without any treatment using 18 cm cell lifters (Corning).

To harvest cells from various phases of the cell cycle, HeLa cells were synchronized by DTB then release and harvested at various time points. S, G2, and G1 cells were released into 37°C DMEM supplemented with 10% FBS (media), then harvested after 2.5 h, 6 h, and 10.5 h, respectively. Early mitosis (P: prometaphase-like) cells were released into media for 5 h then incubated in 50 ng/ml Noc for 3.5 h prior to harvesting by light pipetting. Late mitosis (A: anaphase and telophase) cells were released for 5 h then incubated in 12 ng/ml Noc for 3.5 h prior to collection by light pipetting. Collected cells were washed quickly with media two times then incubated in fresh media for 40 min before harvesting by light pipetting.

### Immunoprecipitation

HeLa cells lysates were prepared by needle shearing as previously described [21], [29]. Cells were lysed in a reducing buffer (10 mM dithiothreitol, 1% SDS, 5 mM EDTA) for 5 min on ice. Cell lysates were diluted 10-fold with RIPA buffer (150 mM NaCl, 25 mM Tris pH 7.5, 1 mM EDTA, 0.5% deoxycholate, 1% NP40) supplemented with 1 mM PMSF, mammalian protease-inhibitor cocktail, 5 mM Na_3_VO_4_, 5 mM NaF, serine-threonine and tyrosine phosphatase inhibitor cocktails, 10 mM NaB (all from Sigma-Aldrich) and 15 U/ml DNase1 (Roche), sheared with a 25 1/2-gauge needle, and precleared with normal rabbit serum bound protein G sepharose beads at 4°C for 1 h. Lysates (2 mg) were immunoprecipitated using anti-NudC G1 goat antibody, followed by blotting with monoclonal anti-Ac-K antibody (Cell Signaling, 1∶1000) and reblotting with the anti-NudC R2 polyclonal antibody in the presence of ReliaBLOT® (Bethyl Laboratories) to reduce background signals. Similarly, histone H3 or α-Tubulin was immunoprecipitated using anti-H3 or α-Tubulin (Sigma-Aldrich) antibody, respectively, followed by blotting with the anti-Ac-K antibody (Cell Signaling) and reblotting with the anti-H3 or α-Tubulin (Genetex) antibody in the presence of ReliaBLOT® (Bethyl) to reduce background signals. The level of acetylation is analyzed by ImageJ (NIH) and presented as a relative ratio of protein acetylation/protein immunoprecipitation or protein input normalized to siLuc or 0 nM apicidin treatment.

### Sequential Immunoprecipitation

Asy and M cells were sheared by a needle as before and lysed for 30 min on ice in lysis buffer (150 mM NaCl, 50 mM Tris, pH 8.0, 5 mM EGTA, 1.5 mM EDTA, 0.1% Triton X-100, 5% glycerol) supplemented with inhibitors as above. Protein concentrations were determined by Bradford assay (Bio-Rad). Asy and M HeLa lysates (5 mg) were first immunoprecipitated overnight at 4°C with 5 μl of the polyclonal anti-Ac-K antiserum (Millipore) then eluted with 1% SDS. The eluates were then diluted to 0.1% SDS with lysis buffer and immunoprecipitated with 4 μl (∼3.5 μg) of the polyclonal anti-NudC G1 antibody then blotted using the NudC R2 antibody. IB for α-tubulin served as a loading control. The level of NudC acetylation is analyzed by ImageJ (NIH) and presented as a relative ratio of immunoprecipitant/Input normalized to Asy.

### Identification of NudC Acetylation Site(s)

Lysates (9 mg) from asynchronous HeLa cells were immunoprecipitated with anti-NudC G1 antibody in lysis buffer supplemented with 10 mM NaB. Immunoprecipitated proteins were resolved by 4–12% SDS-PAGE and stained by GelCode Blue (Thermo Fisher Scientific). The NudC band was excised, digested in-gel with 200 ng modified trypsin (sequencing grade, Promega) for 18 h at 37°C, extracted and analyzed by Nano-LC-MS/MS with on-line desalting using the Nano-LC system (Dionex Corp.). Column effluent was introduced to the mass spectrometer using the Thermo Scientific NanoSpray source fitted with a spray tip from New Objective. Electrospray ion trap mass spectrometry was performed on a linear ion-trap mass spectrometer (LTQ, Thermo Scientific). Proteins were identified by database searching of the fragment spectra against the NCBI non-redundant protein database using Mascot (Matrix Science, London, UK) or Sequest (Thermo).

### siRNA and NudC Acetylation Mutants Transfection

Transfection with control siRNA (siLuciferase) (Dharmacon) [22], siNudC-1 [nt 215–236, AAC ACC TTC TTC AGC TTC CTT] [22] or siGENOME HDAC3-specific siRNA [nt 1334–1352, AAA GCG ATG TGG AGA TTT A] (Dharmacon) [25] was performed by using Oligofectamine (Invitrogen) according to manufacturer's instructions. These sequences were chosen from more than one siRNA targeting sequences and both have been previously shown to be specific and do not elicit off-target effects [25]. For the siRNA depletion followed by rescue experiments, 24 h after siNudC treatment cells were rescued with siRNA-resistant EGFP-hNudC, siRNA-resistant EGFP-hNudC K39R, or siRNA-resistant EGFP-hNudC K39Q for 24 h followed by single thymidine block and release. Silent mutations were introduced to confer siRNA resistance to EGFP-hNudC as a template. siRNA resistance primers: 5′ – G AAC ACG TTC TTT TCC TTC CTG CGA CG – 3′ and 5′ – CG TCG CAG GAA GGA AAA GAA CGT GTT C – 3′. Acetylation mutant constructs were generated by site-directed mutagenesis (Agilent Technologies, Santa Clara, CA, USA) using siRNA-resistant EGFP-hNudC as a template. K39R primers: 5′ – C ATT GAG GAC GGC AGG GTG GTG ACT GTG C – 3′ and 5′ – G CAC AGT CAC CAC CCT GCC GTC CTC AAT G – 3′. K39Q primers: 5′ – C ATT GAG GAC GGC CAG GTG GTG ACT GTG – 3′ and 5′ – CAC AGT CAC CAC CTG GCC GTC CTC AAT G – 3′. The siRNA-resistant EGFP-NudC constructs were transfected using Lipofectamine 2000 (Invitrogen).

### Immunofluorescence Microscopy

HeLa cells were cultured on coverslips and then pre-extracted before fixation. Coverslips were rinsed with 37°C PHEM (60 mM K-PIPES, 25 mM HEPES, 10 mM EGTA, 2 mM MgSO4, pH6.9 with KOH), permeabilized with 0.5% Triton X-100 in PHEM, rinsed with PHEM, and fixed in 4% PFA diluted in PHEM for 20 min. The fixed cells were incubated with antibody blocking solution (0.1 M K-PIPES, 1 mM MgSO4, 1 mM EGTA, 1.83% L-lysine, 1% BSA, 0.1% NaN3, pH7.2 with KOH, pre-saturated with 2% BSA at 4°C), incubated overnight at 4°C with primary antibody [NudC (G1), HDAC3 (N2C3), or β-tubulin (Sigma-Aldrich)], followed by Alexa Fluor conjugated secondary antibody (Invitrogen) for 3 h. To determine NudC and HDAC3 localization on microtubules, HeLa cells were cultured on coverslips, treated with 250 ng/ml Noc for 16 h or 10 μM paclitaxel (Taxol; Sigma-Aldrich) for 1 h, and the coverslips processed as above. For the siRNA depletion and rescue experiments, HeLa cells were cultured on coverslips and treated with siRNA oligos for 48 h with or without rescue for 24 h with EGFP-NudC, EGFP-NudC K39R, or EGFP-NudC K39Q. Following siRNA treatment, cells were synchronized by a single thymidine block (2 mM thymidine for 14.5 h) and released for 8.5 h until harvest to enrich for mitotic cells. The coverslips were fixed with 4% paraformaldehyde at 4°C, permeabilized with 0.5% Triton for 15 min at room temperature, and stained as above with β-tubulin and CREST antiserum. Coverslips were mounted with Gold antifade reagent with DAPI (Invitrogen). Images were acquired using a Nikon TE2000 wide-field microscope system (Nikon Instruments Inc.) and a 40X oil/1.40 NA objective and presented using Adobe PhotoShop CS3 (Adobe Systems Inc.). Spindle widths were measured using NIS-Elements AR 3.1 (Nikon).

### NudC/HDAC3 Protein Association

S, G2, P, A, and G1 HeLa cells were needle sheared as before and protein concentrations were determined as above. Lysates (2 mg) were immunoprecipitated with a monoclonal anti-HDAC3 antibody (Millipore) followed by blotting for NudC with a polyclonal anti-NudC R2 antibody. The association of NudC with HDAC3 is analyzed by ImageJ and presented as a relative ratio of NudC/HDAC3 immunoprecipitation.

### Live-cell Imaging

HeLa cells stably expressing GFP-H2B [30] were seeded on a sterile 35-mm glass-bottomed dish (VWR) or HI-Q^4^ (Nikon), and transfected with siLuc, siNudC or siHDAC3 for 24 h. Images were acquired every 15 min using phase-contrast and FITC filters at 20X magnification for 48 h on a BioStation IM (Nikon). Five to ten image capture points were selected for each treatment, acquired as 3 z-stacks with a step size of 2 µm, and compiled using BioStation IM (Nikon). Images were extracted using NIS-Elements AR 3.0 (Nikon) and presented using Adobe PhotoShop CS3 (Adobe Systems, San Jose, CA, USA) as described previously [27], [30].

### Statistical Analysis

Data were confirmed in multiple independent experiments and expressed as the mean ± SEM. Microsoft Excel 2007 (Microsoft Corp.) was used to plot bar graphs and Student's *t* test was used to calculate the significance for knockdown phenotypes. GraphPad Prism (GraphPad Software Inc.) was used to plot dotplots and one-way ANOVA was used to calculate significance for knockdown and rescue phenotypes. *p* values of less than 0.05 were considered statistically significant.

## Results

### NudC Acetylation and Deacetylation in Mitosis

**Figure 1:**
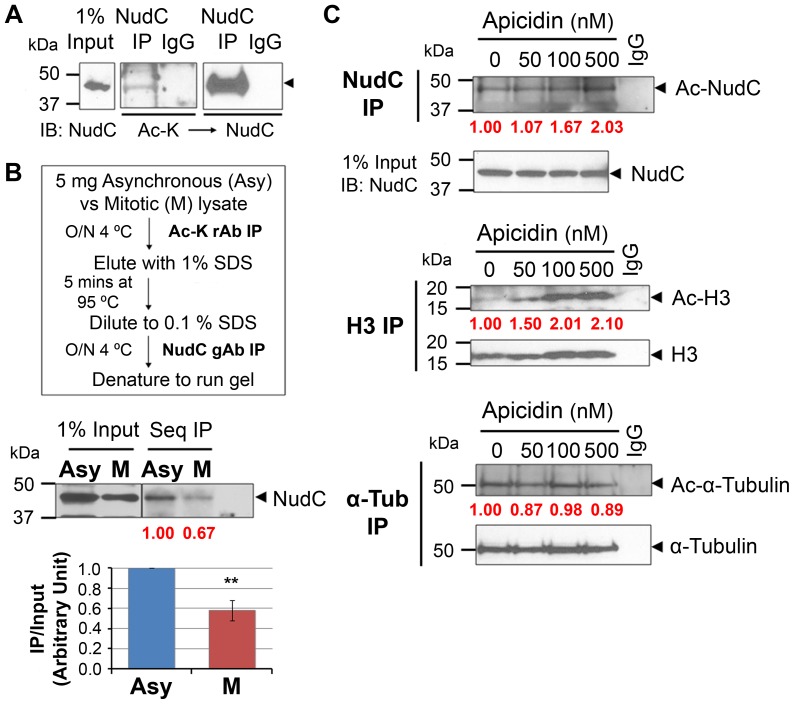
NudC is acetylated in interphase and deacetylated in mitosis. (A) Unperturbed HeLa cell lysates (1 mg) were immunoprecipitated (IP) for NudC under denaturing conditions (used subsequently to examine NudC acetylation) and blotted with anti-acetyl lysine (Ac-K) antibody then reblotted for total NudC. (B) Sequential IP (Seq IP) flow chart. Lysates (5 mg) from asynchronouse (Asy) and mitotic (M) cells are IP'd first with anti-Ac-K antibody followed by IP for NudC then blotted for NudC. NudC acetylation is analyzed by ImageJ as a ratio of NudC IP over Input and normalized to R (fold change in red). Mean ± SEM of three independent experiments. **, p<0.02. (C) Cells were synchronized by a double thymidine block and release (see Figure 4A) then enriched in mitosis with nocodazole (Noc) with or without incubation with increasing concentrations of apicidin. Lysates (2 mg) were IP'd for NudC, histone H3 (H3), or α-tubulin (α-Tub) as in (A), and blotted with anti-Ac-K then reblotted for NudC, H3, or α-Tub, respectively. NudC, H3, and α-Tub acetylation are analyzed by ImageJ as a ratio of Ac-protein over either input (NudC) or protein IP (H3, α-Tub) and normalized to 0 nM Apicidin treatment (fold change in red). Data represent two to three independent experiments.

NudC was identified as an acetylated protein using mass spectrometry of acetylated proteins in mitosis [21]. Treatment with an HDAC inhibitor during mitosis further enhanced NudC acetylation [21], raising the possibility that acetylated NudC may be deacetylated during mitosis. To examine NudC acetylation during cell cycle progression, we first analyzed NudC acetylation in unperturbed asynchronous HeLa cells. Immunoprecipitation (IP) of NudC followed by blotting with anti-acetyl lysine (Ac-K) antibody showed that NudC is acetylated in randomly cycling cells (Figure 1A). Next, we compared NudC acetylation levels in asynchronous (Asy) versus mitotic (M) cells. We immunoprecipitated with Ac-K antibody to enrich for acetylated proteins followed by a second immunoprecipitation with NudC antibody to isolate acetylated NudC (Figure 1B). We found that NudC is acetylated in both Asy as well as M cells. However, NudC acetylation is decreased ∼40% during mitosis, indicating that a significant fraction of NudC is deacetylated in mitosis.

To further examine NudC deacetylation in mitosis, we treated mitotic cells with apicidin, an HDAC2/3 inhibitor [28]. HeLa cells synchronized in mitosis were treated with apicidin for 3.5 h prior to harvesting for NudC immunoprecipitation and blotting for acetylation. We found that increasing concentrations of apicidin resulted in a two-fold increase in the level of NudC acetylation upon 500 nM apicidin treatment (Figure 1C, top), suggesting that NudC is deacetylated during mitosis. Treatment with apicidin did not change total NudC levels as indicated in the input lanes. Acetylation of histone H3 was also increased by two-fold in response to apicidin treatment (Figure 1C, middle), consistent with histone H3 deacetylation being regulated by HDAC2/3 [31], [32]. On the other hand, no change in α-tubulin acetylation was observed (Figure 1C, bottom), consistent with α-tubulin deacetylation being regulated by HDAC6 [33], [34]. Together, these results suggest that NudC is acetylated in HeLa cells, and that a significant fraction of NudC is deacetylated by HDAC2/3 activity in mitosis.

### NudC is Acetylated on K39

We next determined the acetylation sites on NudC by mass spectrometry. Since NudC is more acetylated in asynchronous cells (Figure 1B), unperturbed HeLa cells were used for NudC immunoprecipitation (IP) followed by mass spectrometry using a linear ion trap mass spectrometer (see Figure S1). NudC peptides corresponding to R.^39^KTDFFIGGEEGMAEK^53^.L and R.^38^RKTDFFIGGEEGMAEK^53^.L were observed with a shift of 42 Da. Although lysine acetylation and trimethylation generate a similar shift of 42 Da, the fragmentation behavior is consistent with protein acetylation and an N-terminal amino acid modification. These observations indicate the presence of an acetyl group on K39 in NudC.

### Deacetylated NudC is Required for Proper Mitotic Progression

**Figure 2:**
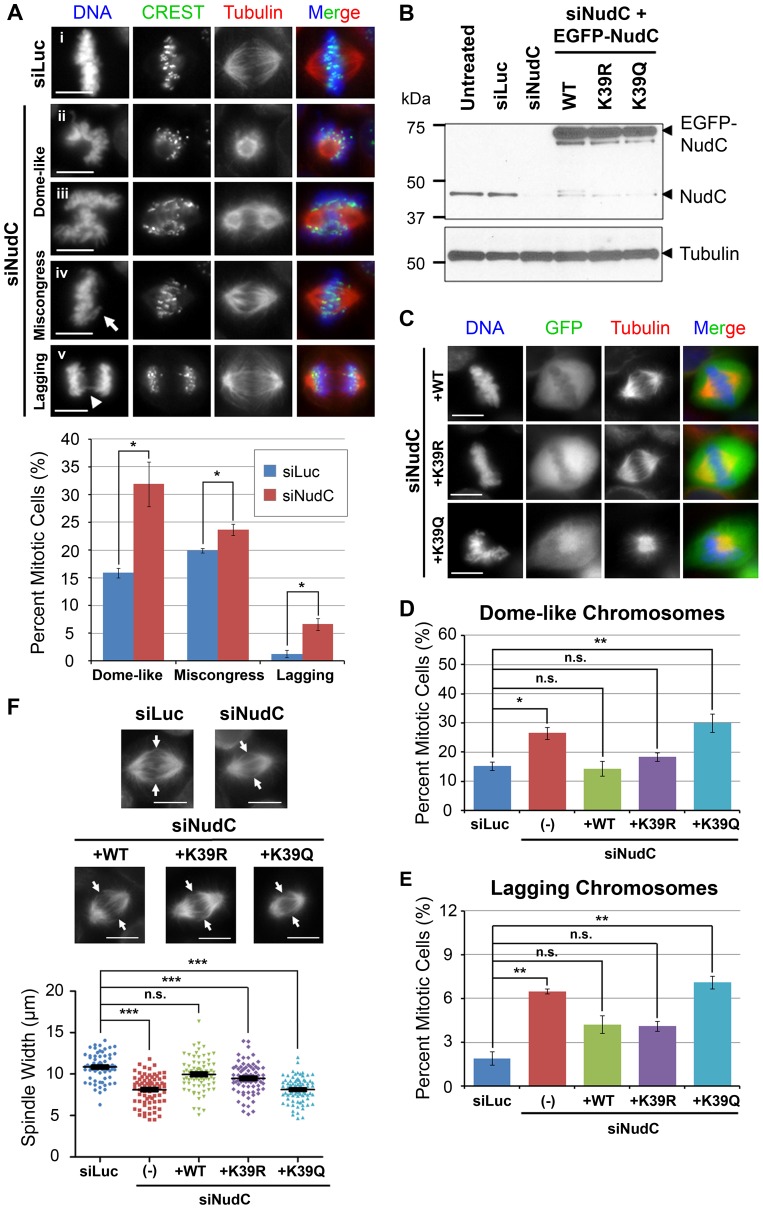
NudC acetylation at K39 regulates mitotic progression. (A) HeLa cells were transfected with siRNA against Luciferase (siLuc; i) or NudC (siNudC; ii–v) 48 h then synchronized by a single thymidine block and release. Cells were stained (left) with CREST (green) to visualize centromeres and β-tubulin (red) for the mitotic spindle and counterstained with DAPI (blue) for DNA. Chromosome phenotypes were observed: “dome-like” configuration of chromosomes that (ii) surround the mitotic spindle or (iii) are involved in lateral, side-on attachments to mitotic spindle; (iv) miscongressed chromosomes at metaphase (arrow); and (v) lagging chromosomes in anaphase/telophase (arrowhead). These chromosome phenotypes were quantified (bottom). Graph shows the mean (± SEM) % from three independent experiments; >200 cells were counted per siRNA oligo. *, p<0.05. (B) Western blot of NudC in NudC knockdown cells without or with EGFP WT, K39R or K39Q reconstitution. HeLa cells were transfected with siLuc or siNudC for 24 h then reconstituted with EGFP-NudC WT, K39R or K39Q lysine mutants for another 24 h. Cells were synchronized by a single thymidine block and release followed by a nocodazole block and release to enrich for mitotic cells. Tubulin was blotted as a loading control. (C) NudC-reconstituted GFP-positive green cells, prepared as in (B), were enriched in mitosis by a single-thymidine block and counterstained with DAPI (DNA; blue). Images represent three independent experiments. Cells in (C) were quantified for “dome-like” configuration of chromosomes (D) and lagging chromosomes (E) relative to siLuc control cells. Graphs show the mean (± SEM) % of three to four independent experiments; >200 cells were counted for each condition. *, p<0.05 and **, p<0.02 by ANOVA. n.s., not significant. (F) HeLa cells were prepared and stained as in (C) and spindle widths (between arrows) were measured. Dotplot shows the mean (± SEM) of ∼70 cells measured for each condition. ***, p<0.0001 by ANOVA. n.s., not significant. Images are representative cells with median spindle widths. All scale bars, 10 µm.

We next examined the significance of K39 acetylation/deacetylation on NudC functions in mitosis by knocking down endogenous NudC followed by expression of K39 mutants of NudC. NudC knockdown resulted in a significant increase in chromosomes that are arranged in a “dome-like” configuration surrounding the mitotic spindle (Figure 2A, ii; and Figure S2B) and chromosomes that are attached in a lateral, side-on manner to the mitotic spindle (Figure 2A, iii), consistent with problems in kineochore-microtubule attachment as previously observed [23]. These siNudC cells are collectively referred to as exhibiting a “dome-like” chromosome configuration of chromosomes surrounding the mitotic spindle. NudC knockdown also increased chromosome miscongression at the metaphase plate (Figure 2A, iv, arrow) as compared to siLuciferase control cells (Figure 2A, i). Quantification showed that ∼30% NudC-deficient cells contained “dome-like” chromosome configuration and ∼25% contained 1–4 miscongressed chromosomes, indicating that ∼55% NudC-deficient cells exhibited chromosome congression errors (Figure 2A, bottom). We also observed a significant increase in lagging chromosomes in ∼6% of NudC-deficient cells relative to control cells during anaphase (Figure 2A, iv, arrowhead; and 2A, bottom), indicating problems with chromosome segregation.

We next examined the ability of the K39 NudC acetylation mutants to rescue the chromosome phenotypes of NudC knockdown cells. siRNA-resistant EGFP-NudC wild-type (WT), acetylation-defective (K39R) and acetylation-mimetic (K39Q) [35], [36] NudC mutants were transfected into NudC-deficient HeLa cells. Western blot analysis confirmed the expression of wild-type and K39 mutants of NudC in NudC-deficient cells (Figure 2B). We then compared the ability of each NudC construct, i.e. NudC-WT, K39R or K39Q, to return the chromosome phenotypes observed in the siNudC cells to the level observed in the control siLuc cells. Wild-type NudC was able to significantly reduce the “dome-like” configuration of chromosomes and the lagging chromosome phenotypes observed in siNudC cells to control levels found in siLuc cells (Figure 2C to E). Similar to wild-type NudC, K39R acetylation-defective NudC was able to reduce both chromosome phenotypes to a level found in siLuc control cells (Figure 2C to E). Thus, both wild-type NudC and the K39R mutant NudC were able to rescue the chromosome phenotypes in NudC-deficient cells. In contrast, K39Q acetylation-mimetic NudC was unable to rescue either chromosome phenotypes, with many cells containing the “dome-like” configuration of chromosomes (Figure 2C to E).

Chromosome alignment and segregation problems may be the result of mitotic spindle defects. We next examined mitotic spindle formation upon NudC depletion. NudC-deficient cells showed a significant decrease in spindle width, suggesting a collapse in the mitotic spindle (Figure 2F). The collapsed spindle may be unable to properly regulate and/or correct kinetochore-microtubule attachments, thus contributing to the miscongressed and lagging chromosome phenotypes observed in these cells (Figure 2A). Wild-type NudC was able to increase the spindle width in NudC knockdown cells to a level observed in siLuc control (Figure 2F). This suggests that wild-type NudC can rescue the spindle defect in NudC-deficient cells. The K39R NudC mutant could only partially rescue the spindle defect, since the difference in spindle width is statistically significant from both the siLuc control cells and the siNudC cells. In contrast, the K39Q NudC mutant was unable to rescue the spindle width defect. Together, these results suggest that during mitosis, deacetylation of NudC on K39 is required for NudC to regulate chromosome alignment, chromosome segregation, and spindle formation.

### NudC Co-localizes with HDAC3 on the Mitotic Spindle

**Figure 3:**
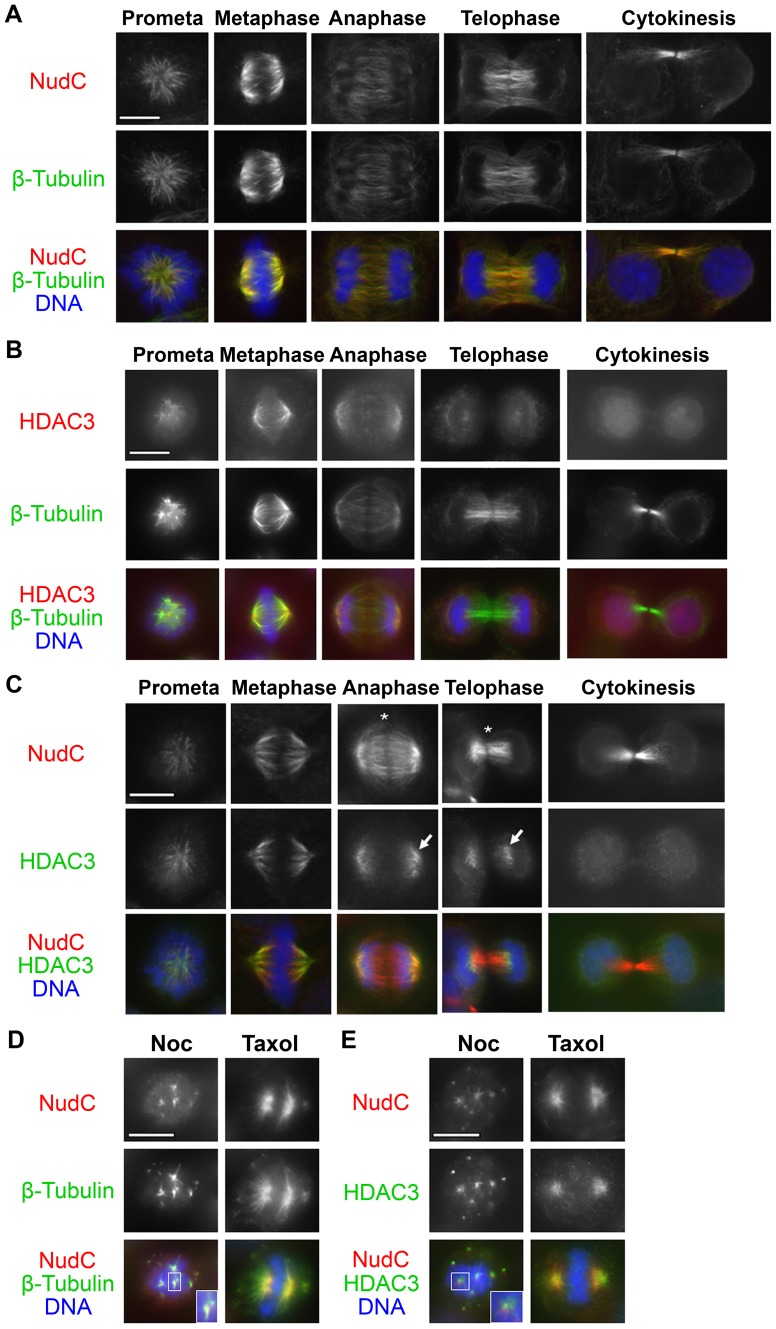
NudC and HDAC3 co-localize in early mitosis. HeLa cells were stained for NudC (A; red) or HDAC3 (B; red) and β-tubulin (A–B; green) then counterstained with DAPI (DNA; blue). (C) Cells were stained for NudC (red) and HDAC3 (green) then counterstained with DAPI (blue). In late mitosis, NudC translocates to the midzone and the midbody (asterisk), whereas HDAC3 remains on the mitotic spindle in anaphase and the minus-end (outer edges) of midzone microtubules in telophase (arrow). Cells were treated with Noc (D) or Taxol (E), stained for β-tubulin (D; green) or HDAC3 (E; green) and NudC (D–E; red), then counterstained with DAPI (DNA; blue). Images represent three independent experiments. All scale bars, 10 µm.

To examine NudC deacetylation during mitosis, we explored whether HDAC2/3 activity plays a role in NudC deacetylation. HDAC2 does not play a role in mitosis [4], [25], [26], while HDAC3 has been shown to localize on the mitotic spindle and regulate spindle stability and chromosome alignment [25]. Thus, we examined whether NudC and HDAC3 co-localize during mitosis by immunofluorescence microscopy. Unperturbed cells undergoing mitosis were stained for NudC or HDAC3 together with β-tubulin. NudC localization on mitotic structures is highly dynamic. NudC first localizes on the mitotic spindle in prometaphase and metaphase, then associates with midzone microtubules during anaphase and telophase, and concentrates at the midbody in cytokinesis (Figure 3A). HDAC3 first localizes on the mitotic spindle with higher concentration towards the two spindle poles in metaphase and anaphase (Figure 3B). HDAC3 concentrates on the minus-ends of the midzone microtubules in telophase and re-localizes in the nucleus during cytokinesis, consistent with its role in regulating gene transcription in the next cell cycle. NudC is found to co-localize with HDAC3 on the mitotic spindle in prometaphase and metaphase (Figure 3C). However, NudC and HDAC3 no longer co-localize well during telophase as NudC translocates to the entire midzone microtubules (Figure 3C, asterisks) while HDAC3 remains associated with the minus-ends of spindle microtubules in anaphase and occupies the outer edge of the midzone microtubules in telophase (Figure 3C, arrows). NudC and HDAC3 also do not co-localize during cytokinesis (Figure 3C). We further treated HeLa cells with nocodazole (Noc) to depolymerize microtubules or taxol to stabilize microtubules (Figure 3D and E). NudC is primarily localized with microtubule spindle remnants in Noc-treated cells or with robust spindle microtubules in taxol-treated cells. Under these conditions, HDAC3 also localizes to the spindle remnants (Figure 3E). Thus, NudC and HDAC3 co-localize on microtubules on the spindle remnants. Together, these studies show that NudC and HDAC3 exhibit dynamic co-localization on the mitotic spindle during early mitosis but not in the later stages of mitosis.

### NudC Associates with HDAC3 in Mitosis

**Figure 4:**
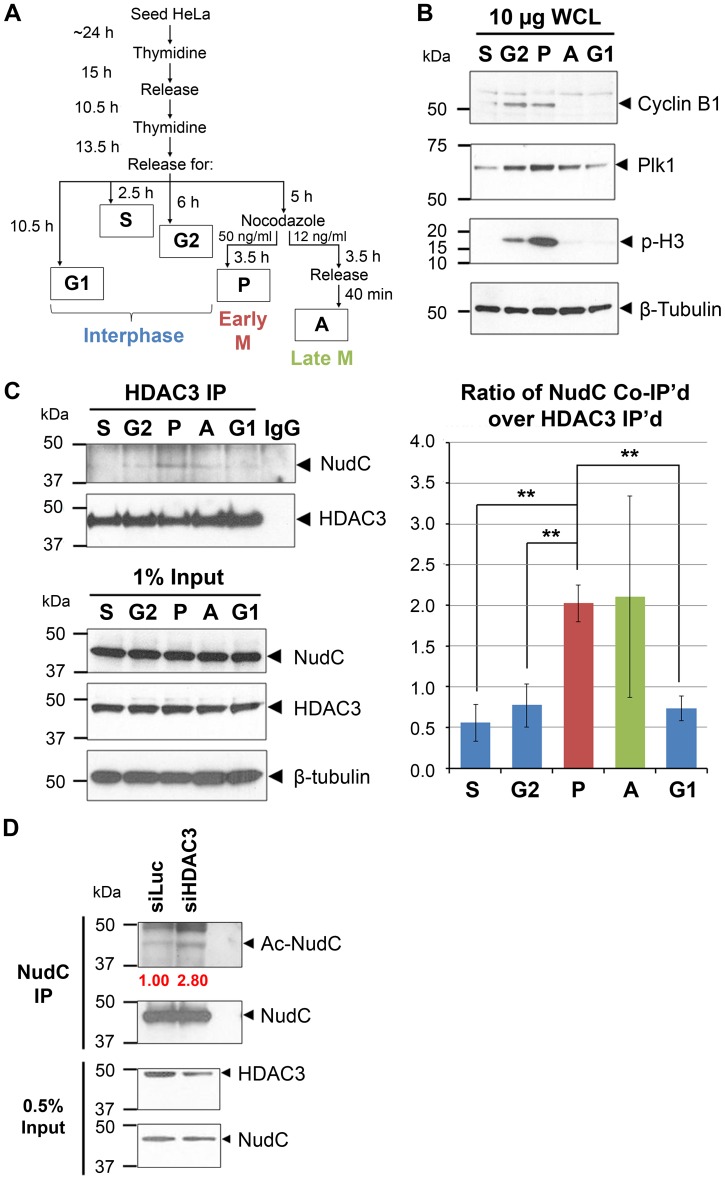
NudC associates with HDAC3 in mitosis and a loss of HDAC3 increases NudC acetylation. (A) Cell cycle synchronization schematic. (B) Whole cell lysates (10 µg) from S, G2, early mitosis (prometaphase-like; P), late mitosis (anaphase and telophase; A), and G1 cell cycle phases are blotted with antibodies as indicated. (C) Lysates (2 mg) prepared as in (B) were immunoprecipitated (IP) for HDAC3 then blotted for NudC followed by HDAC3. IgG, antibody control. β-tubulin was used as a loading control. The association of NudC with HDAC3 is analyzed by ImageJ and presented as a ratio of NudC co-IP over HDAC3 IP. Mean ± SEM of three independent experiments. **, p<.02. (D) Cells were transfected with siRNA against Luciferase (siLuc) or HDAC3 (siHDAC3) for 48 h. Lysates (2 mg) were immunoprecipitated for NudC under denaturing conditions, then blotted for Ac-K and NudC. NudC acetylation is quantified by ImageJ as a ratio of Ac-NudC over NudC and normalized to siLuc (fold changes in red). IgG, antibody control. Data represent three independent experiments.

Since NudC co-localizes with HDAC3 during early phases of mitosis, we further examined whether the two proteins can be found in the same biochemical complex. HeLa cells enriched in different phases of mitosis were prepared as follows. Cells were synchronized using a double thymidine block and release protocol to enrich for G2/M cells as previously described [21], [29]. To further resolve the G2/M population into early and late mitosis, cells were released from double thymidine block for 5 h to reach the G2/M boundary and further treated with either 50 ng/ml nocodazole for 3.5 h to enrich for early mitotic cells (P: prometaphase-like) or 12 ng/ml nocodazole for 3.5 h followed by a 40 min release to enrich for late mitotic cells (A: anaphase and telophase) (Figure 4A). Lysates from cells enriched in G1, S, G2, P or A phases were analyzed by immunoblotting using various mitotic markers (Figure 4B). As expected, both cyclin B1 and polo-like kinase 1 (Plk1) levels increase during G2, peak in early mitosis, and decline in late mitosis, which is consistent with bulk degradation of mitotic regulators by the APC/C proteasome pathway at the metaphase-to-anaphase transition [37]. Further, phosphorylation of histone H3 on serine 10 (p-H3) is maximal in prometaphase and declines by anaphase as expected [14]. Levels of β-tubulin remain essentially unchanged throughout the cell cycle (Figure 4B).

Using this panel of synchronized cell lysates, we found that the levels of NudC and HDAC3 remain the same throughout the cell cycle (Figure 4C, input lanes). However, immunoprecipitation of HDAC3 showed that NudC was mainly associated with HDAC3 during early mitosis (Figure 4C), which may contribute to the deacetylation of NudC observed during mitosis. The variation observed in NudC and HDAC3 association in late mitosis (Figure 4C, right) may be due to difficulty in isolating late mitotic cells as these mitotic phases are very transient, occurring within 25–40 min after release from nocodazole (Figure 4A). The biochemical association of NudC and HDAC3 in prometaphase and metaphase is consistent with their co-localization on the mitotic spindle in early mitosis (Figure 3C).

### HDAC3 Depletion Increases NudC Acetylation

To elucidate whether HDAC3 plays a role in NudC deacetylation, we treated HeLa cells with siRNA against either Luciferase (siLuc; control) or HDAC3 (siHDAC3). Knockdown of HDAC3 led to a 2.8-fold increase in NudC acetylation relative to that in siLuc control cells (Figure 4D). These results are consistent with the increase in NudC acetylation following apicidin treatment (Figure 1C). Together, these results suggest that HDAC3 is involved in regulating NudC deacetylation.

### Depletion of NudC or HDAC3 Results in Overlapping Mitotic Phenotypes

**Figure 5:**
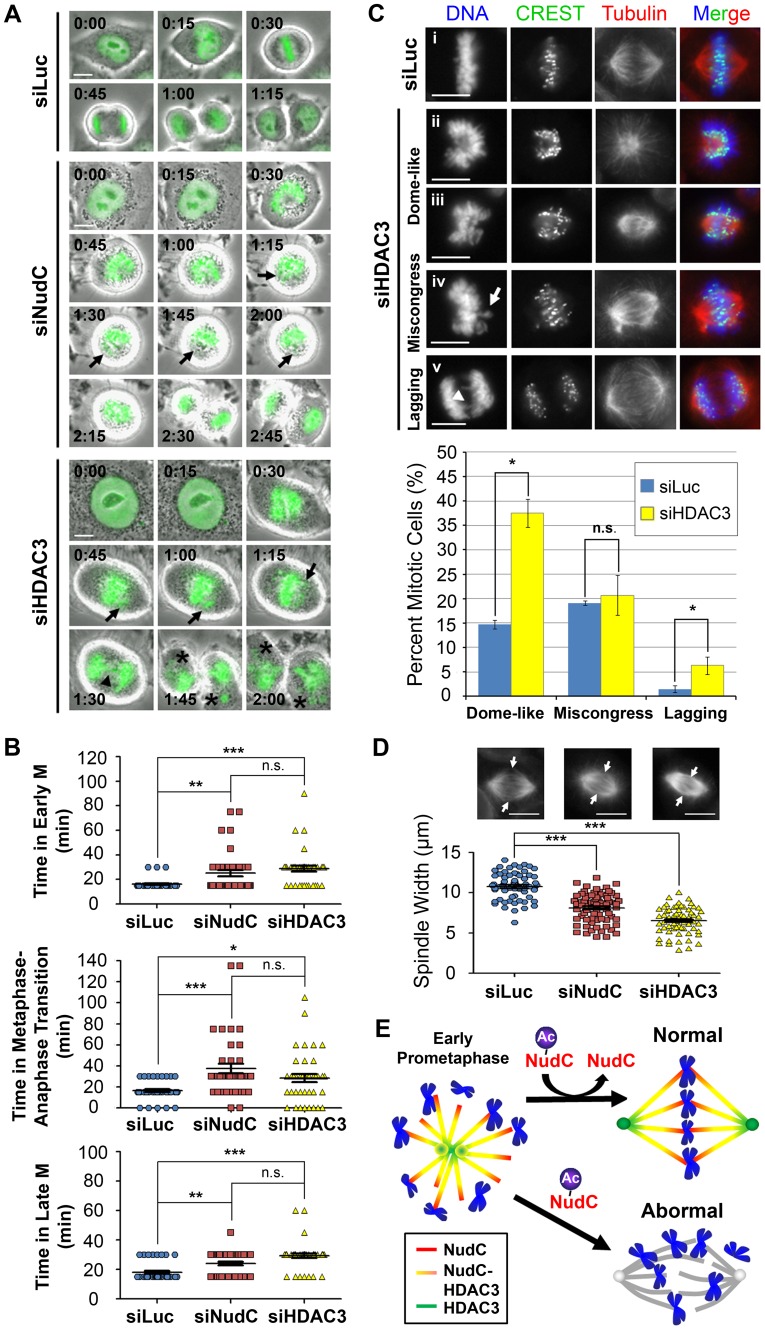
HDAC3 depletion results in similar mitotic defects as NudC depletion. (A) H2B-GFP HeLa cells were transfected with either siHDAC3 or siNudC oligos for 24 h then analyzed by live-cell imaging. Images represent Phase Contrast and FITC (green, DNA). Time for image captured preceding DNA condensation (prophase) is set as 0 h:00 min. Arrow, miscongressed chromosomes. Arrowhead, lagging chromosomes. Asterisks, micronucleation. (B) Based on nuclear/DNA morphology, the timing of mitotic progression was followed for 40 cells for each condition in (A). Graph shows the mean transit time in minutes (min) (± SEM) for the following: Early M (time from prophase to metaphase); Metaphase-to-anaphase transition (time from metaphase to anaphase); Late M (time from anaphase/telophase to cytokinesis). *, p<0.05, **, p<0.01, and ***, p<0.0001 by ANOVA. n.s., not significant. Images are representative of two independent experiments. (C) HeLa cells were transfected with siLuc (i) or siHDAC3 (ii–v) oligos for 48 h then synchronized by a single thymidine block and release. Cells were stained (left) with CREST (green) and β-tubulin (red) then counterstained with DAPI (DNA; blue). Chromosome phenotypes were observed: “dome-like” configuration of chromosome that (ii) surround the mitotic spindle or (iii) are involved in lateral, side-on attachments to the mitotic spindle; (iv) miscongressed chromosomes at metaphase (arrow); and (v) lagging chromosomes in anaphase/telophase (arrowhead). Chromosome phenotypes were quantified (bottom). Graph shows the mean (± SEM) % from three independent experiments; >200 cells were counted per siRNA oligo. *, p<0.05. n.s., not significant. (D) HeLa cells were transfected with siLuc, siNudC or siHDAC3 for 48 h then synchronized by a single thymidine block. Spindle widths (between arrows) were measured and quantified. Dotplot shows the mean (± SEM) of ∼70 cells measured for each condition. ***, p<0.0001 by ANOVA. Images are representative cells with median spindle widths. Scale bars, 10 µm. (D) Model of NudC deacetylation in mitotic progression. NudC deacetylation, likely mediated by HDAC3 activity, is involved in normal mitotic progression. Under conditions where NudC is unable to be deacetylated, such as under HDAC inhibitor treatment, depletion of HDAC3, or expression of K39Q mutant NudC in NudC-deficient cells, cells exhibit abnormal mitosis with a delay in early mitosis and an increase in “dome-like” configuration of chromosomes surrounding a collapsed spindle. The mitotic spindle is shown in different colors to indicate the level of NudC and HDAC3 co-localization: Red, NudC is found in proximity to the chromosomes; yellow, NudC is co-localized with HDAC3; and green, HDAC3 is found in proximity to the spindle poles. Grey, collapsed mitotic spindle. Blue, chromosomes.

As NudC deacetylation is important for mitotic progression, we examined whether knockdown of HDAC3 would produce similar mitotic defects as observed with NudC knockdown. Knockdown of either NudC or HDAC3 did not affect each other's protein levels based on Western blot analysis (data not shown). Using HeLa cells expressing GFP-H2B, we monitored the effects of NudC or HDAC3 knockdown on mitotic progression by live-cell imaging. Transit through early mitosis was timed from DNA condensation at prophase to chromosome alignment at the metaphase plate (Figure 5A). Control siLuc cells traversed early mitosis with an average time of ∼16 min (Figure 5B). In contrast, siNudC and siHDAC3 cells traversed early mitosis with an average time of ∼25 min and ∼29 min, respectively (Figure 5B). These delays in early mitosis are statistically significant when compared to siLuc control cells (Figure 5B). Miscongressed chromosomes (arrows) were detected during metaphase in both siNudC and siHDAC3 cells (Figure 5A), which may contribute to the mitotic delay observed during early mitosis. Timing of cell transit from metaphase into anaphase was also analyzed. A significant delay in metaphase-to-anaphase transition was also observed for siNudC and siHDAC3 cells relative to control cells (Figure 5B). Similarly, a delay in progression through late mitosis from anaphase/telophase to cytokinesis was also observed in both siNudC and siHDAC3 cells relative to control cells (Figure 5A and B; and Figure S2A), with lagging chromosomes observed in these cells (Figure 5A, arrowhead; and Figure S2A, arrowhead). The lagging chromosomes likely contributed to the formation of chromatin bridges between divided daughter cells and resulted in micronucleation in siNudC (Figure S2A, asterisk) and siHDAC3 (Figure 5A, asterisks) cells.

Given the similarity in the timing of mitotic delays, we further compared the chromosome and spindle phenotypes in siHDAC3 versus siNudC cells. Similar to siNudC cells, siHDAC3 cells showed early mitotic defects with chromosomes arranged in a “dome-like” configuration and chromosomes exhibiting lateral, side-on kinetochore attachment to the mitotic spindle (Figure 5C, ii and iii; and 5C, bottom), in agreement with previous observations [25]. Miscongression of chromosomes was also observed in HDAC3 knockdown cells (Figure 5C, iv, arrow; and 5C, bottom), but this did not reach statistical significance relative to siLuc control cells. HDAC3-deficient cells also showed an increase in lagging chromosomes during anaphase relative to control cells (Figure 5C, iv, arrowhead; and 5C, bottom), as also observed in live-cell imaging (Figure 5A), indicating problems with chromosome segregation upon HDAC3 depletion. These mitotic phenotypes in siHDAC3 cells agree with those observed by live-cell imaging (Figure 5A) and are similar to the mitotic phenotypes observed in siNudC cells (Figure 2 and 5A).

We next examined mitotic spindle formation upon HDAC3 depletion. HDAC3-deficient cells showed a significant decrease in spindle width (Figure 5D) as was also observed in NudC-deficient cells (Figures 5D and 2F). The stronger spindle phenotype observed in HDAC3-deficient cells compared to NudC-deficient cells suggests that HDAC3 may have effects on additional targets at the mitotic spindle. Together, these observations suggest that NudC and HDAC3 may function in overlapping pathways in early mitosis.

## Discussion

Our studies show that NudC deacetylation is involved in mitotic progression. Cell cycle analysis indicates that NudC becomes deacetylated in early mitosis likely through its association with the deacetylase HDAC3 (Figure 5E). Deacetylated NudC plays a role in chromosome alignment/segregation and spindle formation. Blocking NudC deacetylation results in a mitotic delay with cells exhibiting chromosome congression errors and spindle abnormality, leading to defective mitosis (Figure 5E).

We envision that NudC is acetylated during interphase and a fraction of NudC is deacetylated in early mitosis. We identified K39 acetylation on NudC using asynchronous HeLa cells without any HDAC inhibitor treatment. Other groups using a variety of human cell types, including MV4-11 acute myeloid leukemic cells, A549 alveolar epithelial cells and Jurkat T cells, together with a 24-h treatment with pan-HDAC inhibitors, identified other potential acetylation sites on NudC (K93, K239, K267) [18] (http://www.phosphosite.org). The differences in acetylation sites identified in our study with those in the other studies may be due to differences in cell types and/or treatment conditions used. It is also possible that the acetylated residues residing on small peptide fragments may not have been detected in our mass spectrometry analysis. Which histone acetyltransferase is involved in the acetylation of NudC is currently unknown. Our studies show that as cells enter mitosis, NudC becomes deacetylated. During early mitosis, NudC not only co-localizes with HDAC3 on the mitotic spindle but NudC is also found in a co-immunoprecipitable complex with HDAC3. Further, NudC acetylation can be increased upon loss of function of HDAC3 by either enzymatic inhibition (apicidin) or protein depletion (siHDAC3). Based on these findings, we suggest that HDAC3 activity is involved in NudC deacetylation. Whether NudC is directly deacetylated by HDAC3 remains to be determined.

NudC deacetylation is required for early mitotic progression. Defects in early mitotic events could result in problems with mitotic progression that lead to defects in later stages of mitosis. We found that under conditions of persistent NudC acetylation as a result of HDAC inhibitor treatment, HDAC3 knockdown, or the expression of K39Q mutant in NudC-deficient cells, cells exhibit defective mitosis with a delay in prometaphase that is likely due to chromosome congression errors and spindle abnormality. In NudC-deficient cells, problems in chromosome congression and spindle function in early mitosis resulted in lagging chromosomes in late mitosis and the formation of chromatin bridges that lead to cytokinesis failure involving micronucleation and multinucleation [22]–[24]. Previous studies by others and our group have shown that cells treated with either apicidin that inhibits HDAC3 [21], [27] or non-selective pan-HDAC inhibitors [4], [21], [38]–[43] undergo aberrant mitosis and cytokinesis failure. Pan-HDAC inhibitors affect many mitotic proteins that play a role in early and late mitosis. Our study suggests that NudC is one of the targets of HDAC3 during early mitosis. Persistent NudC acetylation may contribute to some of the mitotic phenotypes observed with HDAC inhibition.

How NudC acetylation/deacetylation modulates its function in early mitosis is unclear. NudC is a highly conserved protein originally identified as a dynein/dynactin-associated factor involved in transporting nuclei along the germ tube in fungus [44], [45]. During neuronal progenitor cell development in the brain [46], [47] NudC is required for multi-protein transport along axons in neurons [48]–[50]. NudC also plays a role in embryonic cell division in *Pleurodeles waltl* [51], *Caenorhabditis elegans* [22], [52] and *Drosophila melanogaster* [53]. We and others have shown that NudC is localized on major mitotic structures [22], [24], [54] and is involved in regulating spindle formation [54], stability of kinetochore-microtubule attachments [23], and chromosomes congression [23] in early mitosis. These studies raise the possibility that NudC acetylation/deacetylation may affect its ability to associate with the dynein-dynactin complex to regulate microtubule-based mitotic functions.

NudC has been shown to be modified by other posttranslational modifications such as phosphorylation [23], [24]. During early mitosis, NudC is involved in recruiting Plk1 to the kinetochore and Plk1-phosphorylated NudC is important for kinetochore-microtubule attachment and chromosome congression to the metaphase plate [23]. Our studies indicate that NudC K39 acetylation did not interfere with NudC's interaction with Plk1, as NudC K39 mutants can be co-immunoprecipitated with Plk1 (see Figure S3). Whether NudC acetylation affects its phosphorylation by Plk1 remains to be determined.

Several mitotic regulators have also recently been found to be regulated by acetylation/deacetylation, with different consequences on mitotic progression. Aurora B has been found to be acetylated and deacetylated Aurora B exhibits increased kinase activity as compared to acetylated Aurora B [27]. Aurora B associates with HDAC3 at the kinetochore-microtubule interface and this interaction during prometaphase is thought to act as a rheostat to fine-tune Aurora B kinase activity for error correction at the kinetochore-microtubule interface on congressing chromosomes. The microtubule-binding protein, end binding protein EB1, is also acetylated in mitosis and persistent acetylation prevents EB1 from binding to microtubule plus-end tracking proteins (TIPs) [55]. Indeed, deacetylated EB1 shows a more stable interaction with TIPs that leads to efficient and stable kinetochore-microtubule attachments and proper chromosome alignment. How acetylation/deacetylation of these mitotic proteins is regulated awaits further analysis.

Protein acetylation/deacetylation has previously been shown to act as a regulatory switch during cell cycle phase transitions. Acetylation of cyclin A mediates its protein degradation to allow early mitotic progression at prometaphase [6]. On the other hand, acetylation of the spindle checkpoint protein BubR1 blocks its degradation to prevent premature metaphase-to-anaphase transition [7], [56]. Thus, protein acetylation/deacetylation coupled to protein degradation irreversibly regulates mitotic entry as well as mitotic exit. Our studies demonstrate that acetylation/deacetylation of NudC modulates protein functions and provides a new biochemical control mechanism for mitotic progression.

## Supporting Information

Figure S1 **NudC is acetylated at K39.** Lysates (8 mg) from asynchronous HeLa cells were immunoprecipitated (IP) for NudC. The NudC band (red box) was analyzed by ion trap mass spectrometry (ESI-LTQ). HC, heavy chain. LC, light chain. MS/MS spectrum identifies K39 as acetylated in a NudC peptide of ^39^K(Ac)TDFFIGGEEGMAEK^53^.
(TIF)

Figure S2 **NudC knockdown results in multinucleation and micronucleation.** (A) H2B-GFP HeLa cells were transfected with siNudC oligo for 24 h then analyzed by live-cell imaging. Images represent Phase Contrast and FITC (green, DNA). Time for image captured preceding DNA condensation (prophase) is set as 0 h:00 min. Arrow, miscongressed chromosomes. Arrowhead, lagging chromosomes. Asterisk, micronucleation. (B) HeLa cells were transfected with siRNA against NudC (siNudC) for 48 h then synchronized by a single thymidine block and release. Cells were stained with CREST (green) and β-tubulin (red) then counterstained with DAPI (DNA; blue). All scale bars, 10 µm.
(TIF)

Figure S3 **NudC acetylation mutants associate with HDAC3 and Plk1.** HeLa cells were transfected with EGFP-NudC WT, K39R or K39Q lysine mutants for 24 h and synchronized by a single thymidine block and release followed by a nocodazole block and release to enrich for mitotic (M) cells. Lysates (2 mg) were immunoprecipitated (IP) using GFP antibody, blotted for HDAC3 followed by Plk1, and reblotted with GFP for NudC. IgG, antibody control.
(TIF)
