# Supplementary figures and images for: NudC Deacetylation Regulates Mitotic Progression
Source: PLoS One. 2013 Sep 19;8(9):e73841. doi: 10.1371/journal.pone.0073841 (PMC3777959; doi:10.1371/journal.pone.0073841)

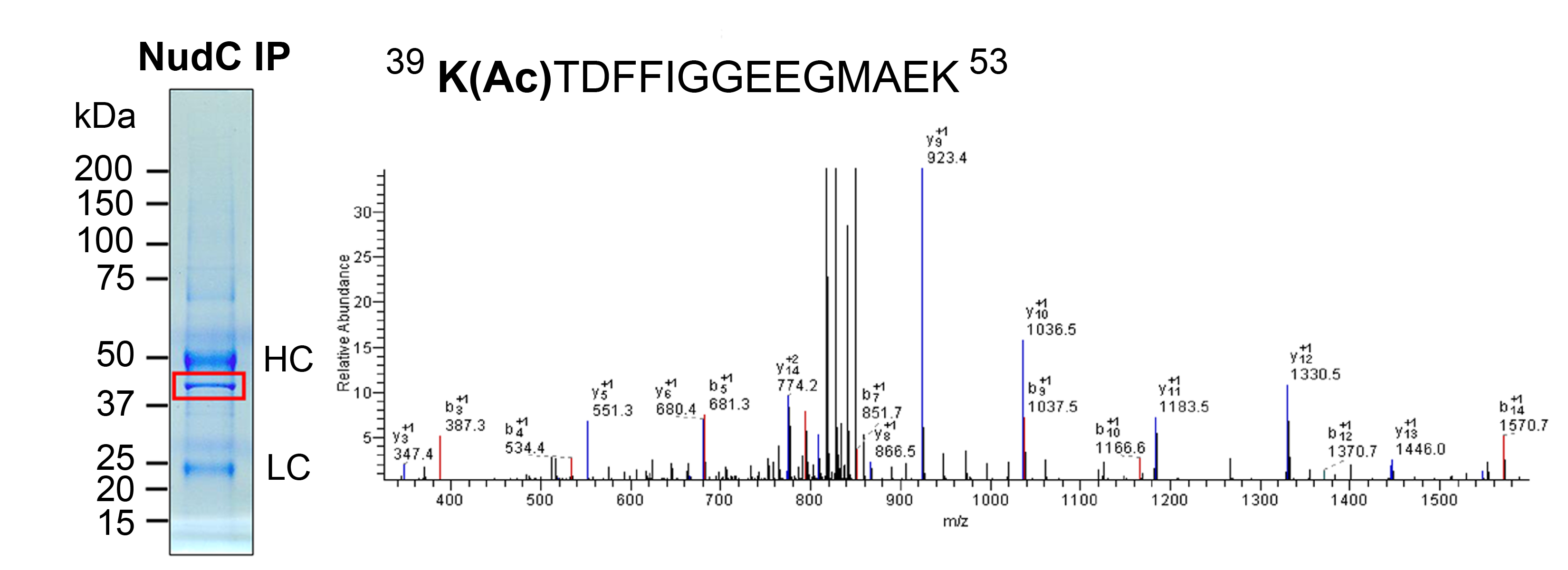

Supplement: Figure S1 — NudC is acetylated at K39. Lysates (8 mg) from asynchronous HeLa cells were immunoprecipitated (IP) for NudC. The NudC band (red box) was analyzed by ion trap mass spectrometry (ESI-LTQ). HC, heavy chain. LC, light chain. MS/MS spectrum identifies K39 as acetylated in a NudC peptide of 39K(Ac)TDFFIGGEEGMAEK53. (TIF) [file pone.0073841.s001.tif]

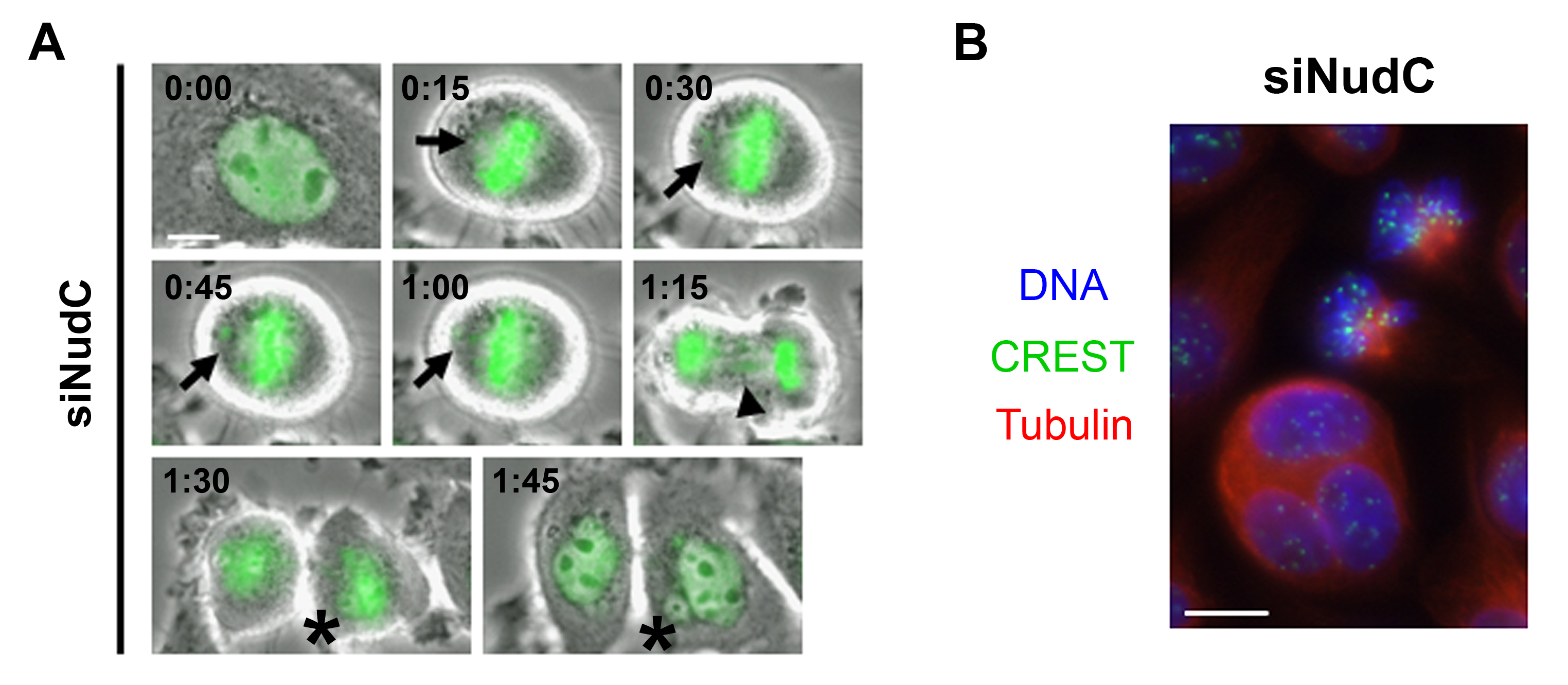

Supplement: Figure S2 — NudC knockdown results in multinucleation and micronucleation. (A) H2B-GFP HeLa cells were transfected with siNudC oligo for 24 h then analyzed by live-cell imaging. Images represent Phase Contrast and FITC (green, DNA). Time for image captured preceding DNA condensation (prophase) is set as 0 h:00 min. Arrow, miscongressed chromosomes. Arrowhead, lagging chromosomes. Asterisk, micronucleation. (B) HeLa cells were transfected with siRNA against NudC (siNudC) for 48 h then synchronized by a single thymidine block and release. Cells were stained with CREST (green) and β-tubulin (red) then counterstained with DAPI (DNA; blue). All scale bars, 10 µm. (TIF) [file pone.0073841.s002.tif]

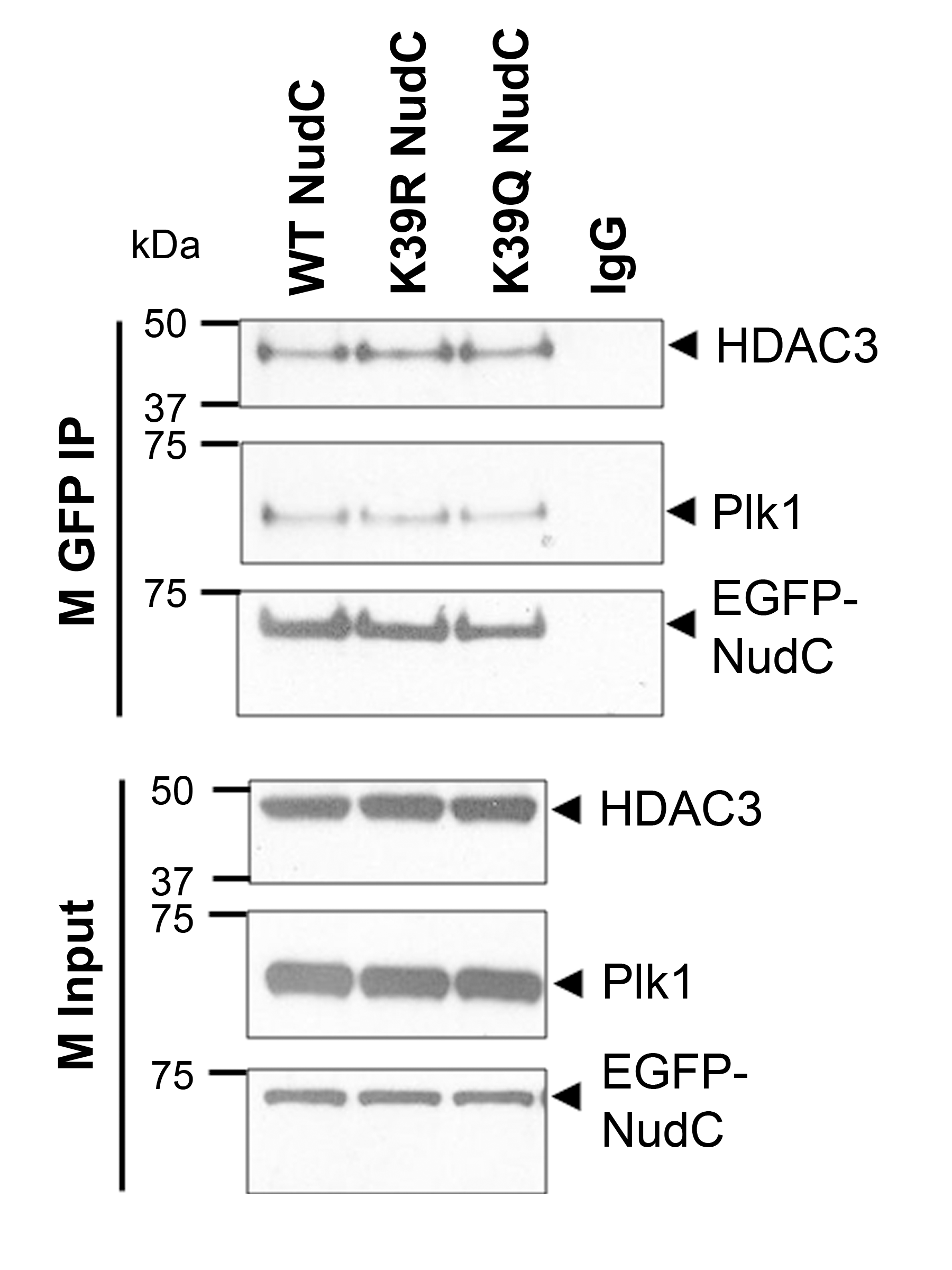

Supplement: Figure S3 — NudC acetylation mutants associate with HDAC3 and Plk1. HeLa cells were transfected with EGFP-NudC WT, K39R or K39Q lysine mutants for 24 h and synchronized by a single thymidine block and release followed by a nocodazole block and release to enrich for mitotic (M) cells. Lysates (2 mg) were immunoprecipitated (IP) using GFP antibody, blotted for HDAC3 followed by Plk1, and reblotted with GFP for NudC. IgG, antibody control. (TIF) [file pone.0073841.s003.tif]
